# Hydrogel Containing Oleoresin From *Copaifera officinalis* Presents Antibacterial Activity Against *Streptococcus agalactiae*

**DOI:** 10.3389/fmicb.2019.02806

**Published:** 2019-12-04

**Authors:** Ana Elisa Belotto Morguette, Briani Gisele Bigotto, Renata de Lima Varella, Gabriella Maria Andriani, Laís Fernanda de Almeida Spoladori, Patrícia Moraes Lopes Pereira, Fabio Goulart de Andrade, Cesar Armando Contreras Lancheros, Celso Vataru Nakamura, Nilton Syogo Arakawa, Marcos Luciano Bruschi, José Carlos Tomaz, Audrey Alesandra Stinghen Garcia Lonni, Gilselena Kerbauy, Eliandro Reis Tavares, Lucy Megumi Yamauchi, Sueli Fumie Yamada-Ogatta

**Affiliations:** ^1^Laboratório de Biologia Molecular de Microrganismos, Departamento de Microbiologia, Centro de Ciências Biológicas, Universidade Estadual de Londrina, Londrina, Brazil; ^2^Programa de Pós-Graduação em Microbiologia, Departamento de Microbiologia, Centro de Ciências Biológicas, Universidade Estadual de Londrina, Londrina, Brazil; ^3^Laboratório de Habilidades Farmacêuticas, Departamento de Ciências Farmacêuticas, Centro de Ciências da Saúde, Universidade Estadual de Londrina, Londrina, Brazil; ^4^Laboratório de Análise Histopatológica, Departamento de Histologia, Centro de Ciências Biológicas, Universidade Estadual de Londrina, Londrina, Brazil; ^5^Laboratório de Inovação Tecnológica no Desenvolvimento de Fármacos e Cosméticos, Departamento de Ciências Básicas da Saúde, Centro de Ciências da Saúde, Universidade Estadual de Maringá, Maringá, Brazil; ^6^Programa de Pós-Graduação em Ciências Farmacêuticas, Departamento de Ciências Farmacêuticas, Centro de Ciências da Saúde, Universidade Estadual de Londrina, Londrina, Brazil; ^7^Laboratório de Pesquisa e Desenvolvimento de Sistemas de Liberação de Fármacos, Departamento de Farmácia, Centro de Ciências da Saúde, Universidade Estadual de Maringá, Maringá, Brazil; ^8^Núcleo de Pesquisa em Produtos Naturais e Sintéticos, Departamento de Física e Química, Faculdade de Ciências Farmacêuticas de Ribeirão Preto, Universidade de São Paulo, Ribeirão Preto, Brazil; ^9^Departamento de Enfermagem, Centro de Ciências da Saúde, Universidade Estadual de Londrina, Londrina, Brazil

**Keywords:** antibiofilm activity, copaiba oil, group B *Streptococcus*, semi-solid formulation, carbomer

## Abstract

*Streptococcus agalactiae* or Group B *Streptococcus* (GBS) remains a leading cause of neonatal infections worldwide; and the maternal vaginal-rectal colonization increases the risk of vertical transmission of GBS to neonates and development of infections. This study reports the *in vitro* antibacterial effect of the oleoresin from *Copaifera officinalis* Jacq. L. *in natura* (copaiba oil) and loaded into carbomer-hydrogel against planktonic and sessile cells of GBS. First, the naturally extracted copaiba oil was tested for the ability to inhibit the growth and metabolic activity of planktonic and sessile GBS cells. The time-kill kinetics showed that copaiba oil exhibited a dose-dependent bactericidal activity against planktonic GBS strains, including those resistant to erythromycin and/or clindamycin [minimal bactericidal concentration (MBC) ranged from 0.06 mg/mL to 0.12 mg/mL]. Copaiba oil did not inhibit the growth of different *Lactobacillus* species, the indigenous members of the human microbiota. The mass spectral analyses of copaiba oil showed the presence of diterpenes, and the kaurenoic acid appears to be one of the active components of oleoresin from *C. officinalis* related to antibacterial activity against GBS. Microscopy analyses of planktonic GBS cells treated with copaiba oil revealed morphological and ultrastructural alterations, displaying disruption of the cell wall, damaged cell membrane, decreased electron density of the cytoplasm, presence of intracellular condensed material, and asymmetric septa. Copaiba oil also exhibited antibacterial activity against established biofilms of GBS strains, inhibiting the viability of sessile cells. Low-cost and eco-friendly carbomer-based hydrogels containing copaiba oil (0.5% – CARB-CO 0.5; 1.0% – CARB-CO 1.0) were then developed. However, only CARB-CO 1.0 preserved the antibacterial activity of copaiba oil against GBS strains. This formulation was homogeneous, soft, exhibited a viscoelastic behavior, and showed good biocompatibility with murine vaginal mucosa. Moreover, CARB-CO 1.0 showed a slow and sustained release of the copaiba oil, killing the planktonic and sessile (established biofilm) cells and inhibiting the biofilm formation of GBS on pre-coated abiotic surface. These results indicate that carbomer-based hydrogels may be useful as topical systems for delivery of copaiba oil directly into de vaginal mucosa and controlling *S. agalactiae* colonization and infection.

## Introduction

*Streptococcus agalactiae* or Group B *Streptococcus* (GBS) remains an important causative agent of potentially fatal neonatal infections (Evangelista and Freitas, 2015; Schrag et al., 2016; Seale et al., 2017; Shabayek and Spellerberg, 2018; Patras and Nizet, 2018). During pregnancy, the maternal vaginal-rectal colonization by GBS may increase the risk of preterm birth and perinatal GBS transmission to newborns (Verani et al., 2010). Overall, 1–3% of the neonates colonized with GBS develop early-onset invasive disease up to 7 days after birth, which is characterized by pneumonia and often progresses to sepsis (Schrag et al., 2016; Kim et al., 2018).

Currently, the prevention strategy for early GBS neonatal infections is based on maternal bacterial screening during the 35–37th weeks of gestation and intravenous intrapartum antimicrobial prophylaxis (IAP) for GBS-colonized pregnant women. Beta-lactams (penicillin and ampicillin) are the first-line antibacterials used for IAP. Alternatively, cefazolin, clindamycin or vancomycin are administered to penicillin-allergic pregnant women with high risk of anaphylaxis (Verani et al., 2010). In fact, IAP has led to a dramatic reduction in the incidence of early GBS neonatal disease in developed countries (Russell et al., 2017; Toyofuku et al., 2017). These preventive measures, however, have not yet been fully adopted in most low- and middle-income countries (Le Doare et al., 2017). Moreover, IAP may have unintended consequences, such as: antimicrobial resistance selection among GBS and non-GBS species; increased risk of neonatal and maternal infection with Gram-negative bacteria or fungal species; changes in the neonatal microbiota that may predispose to short- and long-term health problems and increased risk of development of cerebral palsy in neonates (Patras and Nizet, 2018). Hence, there is a need for affordable and safe alternatives for IAP.

Plants have provided several biologically active compounds, many of which have been proven to possess antimicrobial properties (Kokoska et al., 2018; Singh et al., 2018). Indeed, plant derived-compounds have been used for the treatment of several infectious diseases since ancient times (Kokoska et al., 2018; Singh et al., 2018), and traditional medicine still plays an important role in the management of diseases in many countries (Yuan et al., 2016). In Brazil, copaiba oil is one of the most widely used renewable natural remedy in folk medicine because of its various pharmacological properties (Leandro et al., 2012; Tobouti et al., 2017; Trindade et al., 2018; Arruda et al., 2019). This compound, which is rich in terpenes, is traditionally used to treat a wide range of diseases, including mucosa and skin diseases (wound, psoriasis, dermatitis, and leshmaniasis); respiratory diseases (cough, bronchitis, and pneumonia); and genitourinary diseases (cystitis, syphilis, gonorrhea, and vaginal mucus discharge) (Trindade et al., 2018; Arruda et al., 2019).

Species of *Copaifera* are distributed worldwide, with widespread occurrence in South and Central America. Brazil has the greatest biodiversity of *Copaifera* species, with 26 species and 8 varieties distributed in the Amazon Forest, Cerrado, Caatinga and Atlantic Forest biomes (Costa, 2018). *Copaifera* species were included in the National Policy and Program of Medicinal Plants and Herbal Medicines of the Brazilian Ministry of Health, which establishes guidelines and priorities for the development of actions aimed at ensuring rational use of medicinal plants and herbal medicines (Brazil Ministério da Saúde, 2016).

The antimicrobial activity of copaiba oil or its constituents against different microbial species has been reported previously (Otaguiri et al., 2017; Tobouti et al., 2017; Diefenbach et al., 2018; Kian et al., 2018). Several *Copaifera* species have shown antibacterial activity toward *Streptococcus* species, most of which are commensals or pathogens of human oral cavity (Tobouti et al., 2017; Diefenbach et al., 2018). In a previous study, we reported the antibacterial activity of copaiba oil from *Copaifera multijuga* Hayne against GBS strains. This copaiba oil inhibited both the growth of planktonic cells and metabolic activity of biofilms (Otaguiri et al., 2017).

Herein we described the bactericidal effect of oleoresin from *Copaifera officinalis* (Jacq.) L. (copaiba oil) against planktonic cells and biofilms of GBS strains. This *Copaifera* species, like *C. multijuga*, is one of the most abundant species and main source of copaiba oil marketed in Brazil (Trindade et al., 2018). A carbomer-based hydrogel containing oleoresin from *C. officinalis* that preserves the bactericidal activity against GBS strains with no toxicity to murine cervicovaginal mucosa was also developed. Hydrogels are a three-dimensional cross-linked polymeric network with high content of water in its porous structure (Pal et al., 2009) and have been largely explored for drug delivery, including in the antimicrobial area. Hydrogel formulations can improve bioavailability, mucoadhesion, as well as controlled release of the loaded antimicrobial (Yang et al., 2018).

## Materials and Methods

### Bacterial Strains and Growth Conditions

A total of 9 group B *Streptococcus* strains isolated from vaginal-rectal swabs of women with no clinical evidence of infection were obtained from the bacterial collection of the Laboratório de Biologia Molecular de Microrganismos of the Universidade Estadual de Londrina (UEL), Londrina, Paraná, Brazil. Phenotypic and genotypic characteristics of these strains have been previously described (Otaguiri et al., 2013) and are shown in Table 1. *Streptococcus agalactiae* ATCC 13813 and *Lactobacillus acidophilus* ATCC 4356, *L. acidophilus* LA5 (C Hansen), *Lactobacillus rhamnosus* DR20, *L. rhamnosus* IE203, *Lactobacillus crispatus* HI14C2, *Lactobacillus gasseri* HJ1304, and *Lactobacillus casei* CCT1465 were also included. GBS strains were grown in Tryptic Soy Agar (TSA, Oxoid) containing 5% sheep blood (TSA-blood) for 24 h at 37^*o*^C. *Lactobacillus* strains were grown in Man Rogosa and Shape (MRS, De Man et al., 1960) agar for 48 h at 37^*o*^C. Before the experiments, three colonies of GBS or *Lactobacillus* spp. were transferred to Tryptic Soy Broth (TSB, Oxoid) or MRS broth, respectively, and grown for 18 – 24 h at 37°C. Cells were then centrifuged (13000 rpm, at 25°C, for 3 min) and resuspended in 0.85% NaCl solution (saline) to achieve a turbidity equivalent to 0.5 McFarland standard using the DensiCHECK^TM^ PLUS colorimeter (bioMérieux), which corresponded to approximately 1.0–2.0 × 10^8^ colony forming unit (CFU)/mL (standard bacterial suspension). Each standard bacterial suspension was then diluted in culture medium to achieve the cell density (inoculum) used in each assay. GBS strains were stored in TSB containing 5% sheep blood and 20% glycerol, and *Lactobacillus* spp. were stored in MRS containing 20% glycerol, at −80^*o*^C. The study protocols were approved by the Ethics Committee of UEL (Document 193/12-CEP/UEL). The patients signed a written informed consent form agreeing with this publication.

**TABLE 1:** *Streptococcus agalactiae* susceptibility profile and antibacterial activity of copaiba oil and kaurenoic acid.

**GBS isolate (serotype^1^)**	**Resistance genes^1^**	**Susceptibility profile^1^**		**Copaiba oil**		**Kaurenoic acid**	
				
		**DA**	**E**	**MIC (mg/mL)**	**MBC (mg/mL)**	**MIC (mg/mL)**	**MBC (mg/mL)**
GBS ATCC 13813 (II)	–	S	S	0.03	0.06	0.0125	0.03
GBS 1 (V)	–	S	S	0.03	0.06	0.0125	0.06
GBS 2 (Ia)	–	S	S	0.06	0.12	0.0125	>0.1
GBS 37 (Ia)	–	S	S	0.06	0.12	0.0125	>0.1
GBS 46 (Ia)	*mef* (*A/E*)	S	R	0.03	0.12	0.0125	>0.1
GBS 65 (V)	*erm*(*A*)	R	R	0.03	0.06	0.0125	0.1
GBS 66 (III)	*erm*(*B*)	R	R	0.06	0.06	0.0125	0.1
GBS 84 (V)	–	S	S	0.06	0.06	0.0125	0.1
GBS 89 (Ia)	–	S	S	0.06	0.06	0.0125	0.1
GBS 121 (Ia)	*mef* (*A/E*)	S	R	0.06	0.06	0.0125	0.03

*^1^Genotypic and phenotypic characterization of GBS isolates and erythromycin (E) and clindamycin (DA) susceptibility profile previously described (Otaguiri et al., 2013). MIC, minimal inhibitory concentration; MBC, minimum bactericidal concentration.*

### Oleoresin From *Copaifera officinalis*

Oleoresin from *C. officinalis* (copaiba oil) was acquired from Ferquima Indústria e Comércio de Óleos Essenciais (São Paulo, Brazil). The copaiba oil (batch 165) was collected by direct puncture of trunk tree and its density (0.930 g/mL) and characteristics (viscous and yellow liquid) were described in a technical report.

Mass spectral analyses of copaiba oil were performed at low resolution on a Quattro-LC instrument Micromass (Manchester, United Kingdom) provided with an ESI ion source and a triple quadrupole mass analyzer in full scan mode in positive. Solutions were dissolved in MeOH:H_2_O 4:1 (v/v) and infused into the ESI source at a flow-rate of 5 μL/min and the values of cone voltage and collision energy were optimized, using a Harvard apparatus model 1746 (Holliston, MA, United States) syringe pump.

Kaurenoic acid was obtained from the dried roots of *Sphagneticola trilobata* as described previously (Miranda et al., 2015) and kindly provided by Dr. Nilton Syogo Arakawa (UEL). Hardwickiic acid was acquired from Wuhan ChemFaces Biochemical Co (China). The stock solutions of copaiba oil (10 mg/mL), hardwickiic acid (1 mg/mL) and kaurenoic acid (1 mg/mL) were prepared in 10% dimethylsulfoxide (DMSO, Sigma-Aldrich). DMSO did not exceed 1% in assays.

### Hydrogels

#### Preparation of Hydrogels

Hydrogels without (CARB-BF, base formulation) or with copaiba oil (CARB-CO 0.5 and CARB-CO 1.0) were prepared using the carbomer (Carbopol^®^ 940, Biotec), and the composition of each formulation is shown in Table 2. To prepare the carbomer-based hydrogels, the polymer was dispersed in sterile distilled water with constant stirring (mechanical stirrer, Fisatom 713D) until a homogeneous mixture was obtained. The aminomethyl propanol was added as a neutralizer, and the mixture was stirred until a clear gel formed. Next, copaiba oil 0.5% (w/w) or 1.0% (w/w) was incorporated into the prepared base, yielding CARB-CO 0.5 and CARB-CO 1.0, respectively.

**TABLE 2:** Chemical composition of formulations enriched with copaiba oil.

**Polymer type**	**Formulation Code**	**Components (w/w, except CO%)**					
		**Polymer**	**Allantoin**	**Glycerin**	**CO 0.5**	**CO 1.0**	**DW (q.s.)**
A: Carbopol^®^ 940	CARB-BF	0.6	–	–	–	–	100.0
	CARB-CO 0.5	0.6	–	–	0.5	–	100.0
	CARB-CO 1.0	0.6	–	–	–	1.0	100.0
B: Hydroxyethyl cellulose	HE-CELL-BF	2.0	0.5	2.0	–	–	100.0
	B-CO 0.5	2.0	0.5	2.0	0.5	–	100.0
	B-CO 1.0	2.0	0.5	2.0	–	1.0	100.0
C: Sodium acrylates copolymer and lecithin	ACRY-LEC-BF	2.0	0.5	2.0	–	–	100.0
	C-CO 0.5	2.0	0.5	2.0	0.5	–	100.0
	C-CO 1.0	2.0	0.5	2.0	–	1.0	100.0

*w/w, weight/weight; CO, copaiba oil; BF, base formulation; DW, distilled water. B and C hydrogels did not preserve the antibacterial activity of copaiba oil.*

#### Physical and Chemical Characterization of Hydrogels

The hydrogels were evaluated for color, homogeneity, stability, pH, density and viscosity according to the Brazilian Pharmacopeia guidelines (Brazil Agência Nacional de Vigilância Sanitária, 2010). All assays were performed in triplicate in two independent experiments.

To evaluate the color and homogeneity, 1.5 g of each hydrogel was visually analyzed on a dark background. The maintenance of macroscopic characteristics was evaluated daily by visual analysis after 24 h of formulation for 6 months at 4^*o*^C. For the stability test, 10 g of each hydrogel were placed in a conical and graduated tube, centrifuged at 3200 rpm (Baby I Fanem 206-BL equipment, Brazil) for 30 min at room temperature. The following characteristics were analyzed: phase separation, compact sediment formation, and coalescence.

The pHs of hydrogels were measured using pHmeter (MS Tecnopon Special Equipment Ltda, Brazil). The probe was directly immersed in the formulation. The assays were carried out in triplicate and the pH was determined as the average value between the triplicates. The density was evaluated using a glass pycnometer with 10 mL capacity and temperature monitored at 25 ± 5°C. The ratio between the sample mass and water mass represents the specific density of each sample tested.

To determine the hydrogels viscosity, the samples were arranged in a cylindrical vessel (1.9 cm diameter and 6.3 cm height) fully filled. The measures were performed in a viscometer (DV-III^TM^ + Ultra Brookfield Model, Brazil) at 25 ± 5°C at six different spins (0.50, 0.80, 1.10, 1.40, 1.70, and 2.00 rpm). Apparent viscosity, shear and strain rate data were collected after 90, 45 and 45 s.

The *in vitro* release of copaiba oil from the hydrogel was determined using a modified Franz diffusion cells (Pereira et al., 2013), which consisted of a donor and receptor chambers separated by cellulose acetate membrane with 0.45 μm pore size, previously soaked in diffusion solution. The donor chamber was filled with 5 g of CARB-CO 1.0 or 50 μL copaiba oil. The receptor chamber was filled with 50 mL of purified water *plus* 1.0% DMSO (diffusion solution), and kept under constant magnetic stirring at 37 ± 0.5°C. At different time points (0.5, 1, 2, 3, 4, 6, 8, and 24 h) a volume of 2 mL was withdrawn from the receptor chamber, and replaced with equivalent volume of diffusion solution. The amount of released copaiba oil was monitored by spectrophotometry (Synergy^TM^ HT, BioTek) at 300 nm. The *in vitro* release was calculated using a copaiba oil standard calibration curve obtained with different concentrations (0.0009 mg/mL to 1.0 mg/mL, R_2_ 0.998). All the experiments were performed, at least, in three replicates. To investigate the release mechanism, the data generated by the release study were fitted to the general release equation (Korsmeyer et al., 1983; Bruschi, 2015), according to equation (1).

(1) M/tM∞=(k.t)n

Where Mt/M∞ is the amount of copaiba oil released at time t; k is a constant incorporating structural and geometrical characteristics of the system; and n is the release exponent that may indicate the mechanism of copaiba oil release.

#### Effect of CARB-CO 1.0 Hydrogel on Murine Cervicovaginal Mucosa Histology

Female BALB/c mice (8–12 weeks old) were used in this assay. To synchronize the females’ estrous cycle in the proestrus period, 100 μL of β-estradiol (5 mg/mL, Sigma-Aldrich) suspension in sesame oil was administered intraperitoneally 24 h prior applications of hydrogels. The hydrogels (20 μL) were applied once a day into the cervicovaginal mucosa using a sterile swab (Fernández-Romero et al., 2012) during 14 days. Mice treated with CARB-BF or 0.15 M phosphate-buffered saline pH 7.2 (PBS) were used as control. After 24 h and 14 days of hydrogels application, mice were euthanized and the cervicovaginal mucosa was surgically excised. The tissues were fixed in Bouin’s solution and embedded in paraffin before preparing tissue sections of 6 μm, using a microtome (Slee, Mainz, Germany). The sections were stained using hematoxylin and eosin (H&E) and morphological analyses were performed on a light microscope (Motic, Xiamen, China). This study protocol was approved by the Ethics Committee for the Use of Animals of Universidade Estadual de Londrina (Document 127/2017-CEUA/UEL).

### Antibacterial Activity of Copaiba Oil and Hydrogels Containing Copaiba Oil Against Planktonic GBS Cells

#### Determination of Minimal Inhibitory and Bactericidal Concentration of Copaiba Oil

Minimal inhibitory concentration (MIC) of copaiba oil and the acid diterpenes (kaurenoic acid and hardwickiic acid) against planktonic cells was determined by broth microdilution assay according to the Clinical and Laboratory Standard Institute (CLSI, 2015) guidelines. One hundred microliter-aliquots of GBS and *Lactobacillus* spp. (5 × 10^5^ CFU/mL) were added to the wells of 96-wells U-bottom microtiter plates (Techno Plastic Products, Switzerland) containing twofold serial dilutions of copaiba oil (0.001 to 1.0 mg/mL) or acid diterpenes (0.0001 to 0.1 mg/mL) in TSB and MRS, respectively. Wells containing medium or medium *plus* DMSO 1%, and wells without bacteria were used as growth and sterility control, respectively. MIC was determined at total inhibition of bacterial growth after 24 h- (GBS) or 48 h- (*Lactobacillus* spp.) incubation at 37^*o*^C compared to untreated cells.

To determine the minimal bactericidal concentration (MBC), the content from the wells (10 μL) showing no growth were inoculated on TSA-blood or MRS agar plates (Miles et al., 1938). After 24 or 48 h of incubation at 37°C, MBC was defined as the lowest concentration that resulted in absence of growth of treated bacteria compared to untreated bacteria. The assays were performed in triplicate in two independent experiments.

#### Antibacterial Activity of Copaiba Oil Hydrogels

One hundred microliter-aliquots of planktonic cell suspensions (5 × 10^5^ CFU/mL) of all tested GBS strains were spread on the surface of TSA plates. Then, 200 μL of each hydrogel (CARB-BF, CARB-CO 0.5 or CARB-CO 1.0) were spread over the bacterial inoculum and CFU counts were determined after incubation for 24 h at 37°C. The hydrogel containing copaiba oil which promoted total GBS growth inhibition, compared to the untreated control, was selected for further assays. The test was performed in triplicate in two independent experiments.

#### Time-Kill Kinetics of Copaiba Oil and CARB-CO 1.0 Hydrogel

For time-kill kinetics analysis, planktonic cells of GBS ATCC 13813, GBS 66 [resistant to erythromycin and clindamycin, *erm*(B)^+^] and GBS 121 [resistant to erythromycin, *mef*(A/E)^+^] strains at a cell density of 5 × 10^5^ CFU/mL were incubated in presence of copaiba oil (at MIC and MBC values), CARB-BF or CARB-CO 1.0 hydrogel at 37^*o*^C. At determined time points (0, 0.5, 1, 2, 4, 8, 12 and 24 h), 20 μL-aliquots were removed and serially diluted 10-fold in PBS. Ten microliter-aliquots were inoculated on TSA-blood plates and CFU counts were determined after 24 h-incubation at 37°C (Miles et al., 1938). The assay was performed in triplicate in two independent experiments. The means of CFU counts were plotted as log_10_ CFU *versus* time (h).

#### Transmission Electron Microscopy (TEM) Analysis

Morphological and ultrastructural changes provoked by copaiba oil (at 0.5 × MIC, MIC, and MBC) on planktonic GBS cells after 2 h of treatment were analyzed by TEM. Bacterial cells were fixed at room temperature for 2 h with 2.5% glutaraldehyde in 0.1 M sodium cacodylate buffer, pH 7.4; followed by post-fixation in 1% OsO_4_ in cacodylate buffer containing 0.8% potassium ferrocyanide and 5 mM CaCl_2_ for 2 h. The cells were then dehydrated in a graded series of acetone and embedded in Epon resin for 72 h at 60°C. Ultrathin sections were obtained with a Leica ultramicrotome and grids containing the sections were stained with 5% uranyl acetate and lead citrate for further observation in a JEOL JEM-1400 Electron Microscope at 80 kV.

### Antibacterial Activity of Copaiba Oil and CARB-CO 1.0 Hydrogel Against Sessile GBS Cells

#### Biofilm Formation

Biofilms of all GBS strains were formed on polystyrene, flat-bottom 96-well microtiter plates (Techno Plastic Products, Switzerland) using a procedure described by Otaguiri et al. (2017). Briefly, 20 μL of standard bacterial suspension were placed in each well containing 180 μL of TSB, and the plates were incubated statically for 24 h at 37^*o*^C. After incubation, the medium was aspirated off and non-adherent cells were removed by washing carefully with PBS (once).

#### Antibacterial Activity of Copaiba Oil Against Established GBS Biofilm

Biofilms were formed as described above, and after removing non-adherent cells, fresh TSB (200 μL) containing different concentrations of copaiba oil (0.03 to 1.0 mg/mL) or diterpene acids (0.006 to 0.1 mg/mL), was added to the wells, and the plates were incubated for 24 h at 37^*o*^C. Plant products-free wells and biofilm-free wells were included as controls. The metabolic activity of treated sessile cells was compared to untreated cells using the 2,3-bis(2-methoxy-4-nitro-5-sulfo-phenyl)-5-[(phenylamino)carbonyl]-2H-tetrazolium hydroxide (XTT)-reduction assay (Shafreen et al., 2011). Two hundred microliters of XTT-menadione [0.5 mg/mL XTT, 1 mM menadione (Sigma-Aldrich)] were added to each well, and the plates were incubated in the dark for 90 min at 37^*o*^C. The supernatants were transferred to a new microtiter plate, and the optical density (OD) was measured at 490 nm with a microtiter plate reader (Synergy^TM^ HT, BioTek). The assay was performed in quintuplicate in two independent experiments. Sessile minimal inhibitory concentrations were determined at 80% inhibition (SMIC_80_) compared to plant products-free control wells.

#### Antibacterial Activity of CARB-CO 1.0 Hydrogel During GBS Biofilm Formation

One hundred microliters of CARB-CO 1.0 and CARB-BF hydrogels were spread on the surface of flat-bottom 24-well plates (Techno Plastic Products). A 40 μL-aliquot of standard bacterial suspension was placed in each well containing 360 μL of TSB, and the plates were incubated statically for 24 h at 37^*o*^C. After incubation, the medium was aspirated and non-adherent cells were removed by washing (once) carefully with PBS. The biofilms were gently scraped with a sterile tip and vigorously homogenized in PBS pH 7.2 (400 μL). Twenty microliter-aliquots were removed, serially diluted (10-fold) in PBS and 10 μL of each dilution were inoculated on TSA-blood plates. CFU counts were determined after 24 h-incubation at 37°C. Wells containing medium, and wells without GBS cells were used as growth and sterility control, respectively. Experiments were carried out in duplicate in two independent experiments.

#### Microscopy Analyses

Morphological alterations provoked by copaiba oil and CARB-CO 1.0 hydrogel on biofilm of GBS were analyzed by scanning electron microscopy (SEM) and confocal laser scanning microscopy (CLSM). An inoculum of 1.0 × 10^7^ cells was prepared in 1 mL of TSB.

For SEM, the surface of polystyrene strips (0.5 cm^2^) was previously loaded with CARB-CO 1.0 and CARB-BF hydrogels, placed in wells of 24-well tissue cultures plates and then immersed in TSB containing the inoculum. All systems were incubated statically at 37°C for 24 h. Treated and untreated-control biofilms were fixed with 2.5% (v/v) glutaraldehyde in 0.1 M sodium cacodylate buffer pH 7.2 at room temperature for 4 h, and post-fixed in 1% OsO_4_ for 2 h. The cells were dehydrated with a series of ethanol washes (30, 50, 70, 90, and 100%), critical-point dried in CO_2_, coated with gold and observed in a Shimadzu SS-550 scanning electron microscope.

For CLSM, TSB containing the inoculum was added on CELLview^TM^ cell culture dish with glass bottom (Greiner Bio One, Brazil) to form biofilms. After 24 h-incubation at 37^*o*^C, the biofilms were gently washed (once) with PBS pH 7.2 before adding fresh 1.0 mL TSB containing copaiba oil at SMIC, CARB-CO 1.0 and CARB-BF, and the systems were incubated for further 24 h. Biofilms were washed (once) with sterile saline and incubated in 1 mL of PBS containing 60 μM SYTO^®^ 9 (green-fluorescent dye that labels live and dead bacteria) and 60 μM propidium iodide (red-fluorescent dye that selectively labels bacteria with permeable/damaged membranes) from the LIVE/DEAD^TM^ *Bac*Light^TM^ kit (Thermo Fischer Scientific, Brazil) at 4^*o*^C for 15 min in the dark. After washing and drying, the treated and untreated biofilms were observed in a confocal laser scanning microscope [objective type: HC PL APO CS2 63x/1.40 oil; magnification: 63x; zoom: 1.03; numerical aperture: 1.40 (Leica Microsystems, Germany)] with the excitation and emission wavelengths, respectively: 483 and 500 nm for SYTO^®^ 9 and 490 and 635 nm for propidium iodide, both with long-pass filter.

### Statistical Analysis

Data were analyzed using the software GRAPHPAD Prism version 6.0 (GRAPHPAD Software, San Diego, CA, United States). Kruskal-Wallis and Dunn’s’ multiple comparison *post hoc* test were used to analyze MIC and MBC values and time-kill curves; and two-way ANOVA and Bonferroni *post hoc* test were used to analyze biofilm data. *P* values less than 0.05 were considered significant in all cases.

## Results

### Oleoresin From *Copaifera officinalis* Exhibits Bactericidal Activity Against Planktonic GBS Strains Without Affecting *Lactobacillus* Species

**FIGURE 1:**
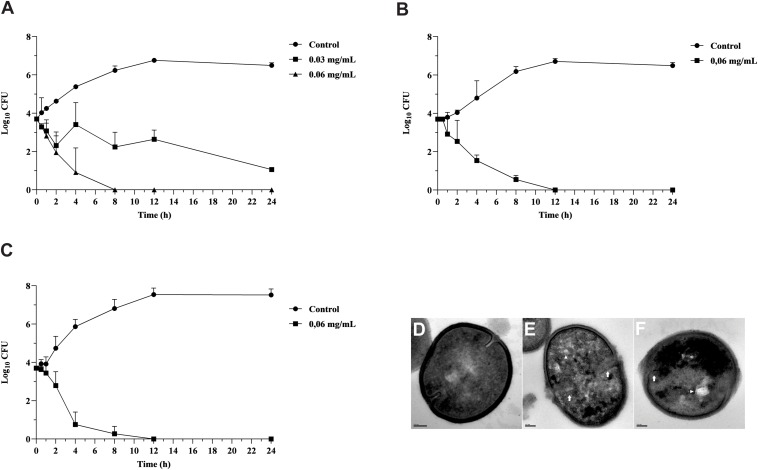
Antibacterial effect of oleoresin from *Copaifera officinalis* on growth and morphology of planktonic cells of *Streptococcus agalactiae*. Time-kill curve of *S. agalactiae* ATCC 13813 **(A)**, erythromycin and clindamycin-resistant GBS 66 strain **(B)**, and erythromycin-resistant GBS 121 strain **(C)** in presence of copaiba oil. Bacteria were incubated with copaiba oil at MIC (0.03 or 0.06 mg/mL) and MBC (0.06 mg/mL) for 24 h at 37°C and the CFU counts per 10 μL were determined at specified time points. GBSs without treatment were used as growth control. Values are mean ± standard deviation of two experiments. Transmission electron microscopy images of untreated *S. agalactiae* ATCC 13813 exhibiting typical spherical morphology, continuous cell wall and regular electron density **(D)**. The treatment with copaiba oil at MIC **(E)**, and MBC **(F)** for 2 h resulted in disruption of cell wall (white arrow), intracellular condensed material (arrowhead), and electron density decreasing. Bar: 100 nm.

Copaiba oil inhibited the growth of planktonic cells of all GBS strains, including those resistant to erythromycin and clindamycin exhibiting different mechanisms of antimicrobial resistance. MIC values were 0.03 and 0.06 mg/mL and MBC values were 0.06 and 0.12 mg/mL, indicating bactericidal activity (Table 1). There was no significant difference (*P* > 0.05) between MIC and MBC values for all strains, therefore the reference strain *S. agalactiae* ATCC 13813, and the clinical strains GBS 66 [resistant to erythromycin and clindamycin, *erm*(B)^+^] and GBS 121 [resistant to erythromycin, *mef*(A/E)^+^] were selected to evaluate the copaiba oil time-kill kinetics (Figure 1). Copaiba oil at MIC (0.03 mg/mL) inhibited the growth of the reference strain and around 2 log_10_-reduction in CFU counts was observed after 24 h of treatment, compared to initial inoculum. At MBC (0.06 mg/mL), no CFU was detected after 8 h (Figure 1A). The CFU counts of GBS 66 (Figure 1B) and GBS 121 (Figure 1C) strains were zero after 12 h of treatment with copaiba oil at MIC/MBC (0.06 mg/mL for both strains). These results supported the bactericidal activity of oleoresin of *C. officinalis* against GBS.

Untreated control cells exhibited typical spherical morphology showing a continuous cell membrane and cell wall, and homogeneous electron density of the cytoplasm (Figure 1D). Conversely, GBS treated with MIC (Figure 1E) and MBC (Figure 1F) values of copaiba oil presented remarkable morphological and ultrastructural alterations, exhibiting disruption of the cell wall, decreased electron density of the cytoplasm, presence of intracellular condensed material, and asymmetric septa.

We also evaluated the effect of *C. officinalis* oil on the growth of *L. acidophilus, L. casei, L. rhamnosus*, *L. crispatus*, and *L. gasseri*, commensal members of the human vaginal microbiota (Hickey et al., 2012). Even at the highest concentration (1.0 mg/mL), copaiba oil had no inhibitory effect on the growth of these bacteria. Despite these results, we cannot rule out the possibility that copaiba oil may have an inhibitory effect on the growth of other members of the human vaginal microbiota.

### Oleoresin From *Copaifera officinalis* Inhibits the Established Biofilm of GBS

**FIGURE 2:**
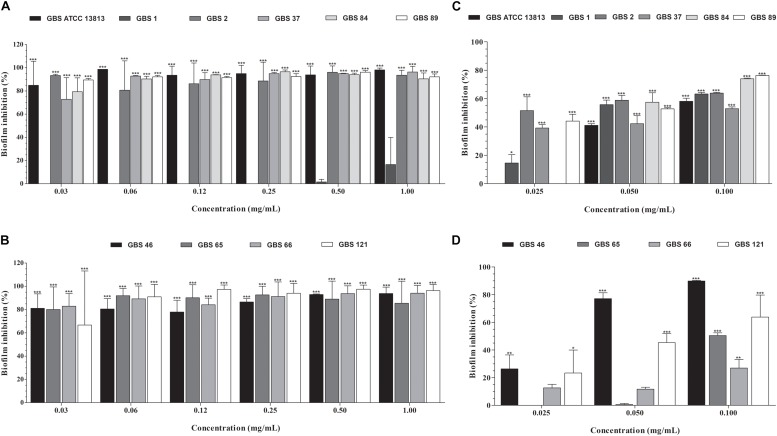
Inhibitory activity of oleoresin from *C. officinalis* **(A,B)** and kaurenoic acid **(C,D)** on *Streptococcus agalactiae* biofilms. Established biofilms (24 h) of erythromycin- and/or clindamycin-susceptible **(A,C)** and -resistant **(B,D)** GBS strains were treated with different concentrations of copaiba oil or kaurenoic acid, and the metabolic activity of sessile cells was assessed by XTT-reduction assay. Values are mean ± standard deviation of two experiments. The asterisks indicate a significant reduction of metabolically active sessile cells treated with copaiba oil compared to untreated cells (^∗^*P* < 0.05, ^∗∗^*P* < 0.01, and ^∗∗∗^*P* < 0.001).

All GBS strains were capable to form biofilm on polystyrene surface (Figure 2). Except for the GBS 1 strain (susceptible to penicillin, erythromycin, and clindamycin), copaiba oil inhibited the established biofilms of all GBS strains at all the concentrations tested (Figures 2A,B). Overall, the percentage reduction of metabolic activity of biofilm (sessile) cells ranged from 72.8% to 98.6%, with SMIC_80_ values ranging from 0.06 to 0.5 mg/mL.

The viability of the reference GBS strain within the biofilms was also analyzed by CLSM after treatment of the cells with a combination of the two fluorescent nucleic acid dyes, SYTO^®^ 9 and propidium iodide. A well-structured biofilm consisting of several aggregates of viable cells (green-fluorescent) was observed in untreated (control) GBS after 24 h-incubation; and the three-dimensional reconstruction of the untreated biofilm showed that these cellular aggregates were organized in a 3.5-μm-thick (see Figures 5A1,A2). In contrast and consistent with the XTT-reduction assay data, a significant increase in red-fluorescent cells was observed in copaiba oil-treated biofilm (see Figures 5B1,B2), reflecting dead bacteria with damaged membranes.

### Kaurenoic Acid, but Not Hardwickiic Acid, Exhibits Antibacterial Effect Against Planktonic and Sessile GBS Cells

Previous studies reported the main phytochemical components of different *Copaifera* species, which may vary according to the trees of the same species from different regions and seasonality (Cascon and Gilbert, 2000; Barbosa et al., 2012; Trindade et al., 2018; Arruda et al., 2019). Overall, a mixture of sesquiterpenes (volatile fraction) and acid diterpenes (non-volatile resin fraction) was found in copaiba oil from different *Copaifera* species (Santos et al., 2008b; Trindade et al., 2018; Arruda et al., 2019). Moreover, many of the biological activities of copaiba oil from different *Copaifera* species have been attributed to the acid diterpene content (Abrão et al., 2015; Vargas et al., 2015; Kian et al., 2018; Trindade et al., 2018; Arruda et al., 2019; Dalenogare et al., 2019). Particularly, kaurenoic acid, hardwickiic acid and copalic acid have been described as main acid diterpene of *C. officinalis* oleoresin (Santos et al., 2008b; Trindade et al., 2018; Arruda et al., 2019; Dalenogare et al., 2019). In light of these data, we performed a mass spectral analyses of the copaiba oil and confirmed the presence of copalic acid [*EIMS* m/z *305.24 (M+)]*, kaurenoic acid *[EIMS* m/z *341.26 (M + K)]*, and hardwickiic acid *[EIMS* m/z *317.22 (M+)]* (Supplementary Figure S1).

Hardwickiic acid did not inhibit the planktonic growth and biofilm of any GBS strains at the tested concentrations (data not shown). Conversely, kaurenoic acid inhibited the growth of planktonic cells of all GBS strains, displaying MIC and MBC values ranging from 0.013 to 0.03 mg/mL and 0.03 to >0.1 mg/mL, respectively. Compared to the MIC and MBC values of copaiba oil, there were no significant differences among them (*P* > 0.05, Table 1), indicating that kaurenoic acid may be one of the active phytochemical of *C. officinalis* oleoresin toward GBS strains. In addition, kaurenoic acid decreased the metabolic activity of established biofilms of all GBS strains in at least one concentration tested. Among erythromycin- and clindamycin-susceptible GBS strains (Figure 2C), the reduction in metabolic activity ranged from 41.1% to 58.8% and 52.9% to 76.3% in presence of 0.05 and 0.10 mg/mL kaurenoic acid, respectively. Among resistant GBS strains (Figure 2D), biofilms of GBS 65 and GBS 66 were inhibited only at 0.10 mg/mL concentration, displaying a reduction of 50.4% and 26.9%, respectively. For GBS 46 and GBS 121 biofilms, a dose-dependent inhibitory effect of kaurenoic acid was observed. Compared to those results obtained with copaiba oil, the antibiofilm activity seems to depend on a synergistic interaction between the components of the oleoresin.

### Carbopol^®^-Based Hydrogel Containing 1.0% Copaiba Oil Preserves the Antibacterial Activity Against GBS

**FIGURE 3:**
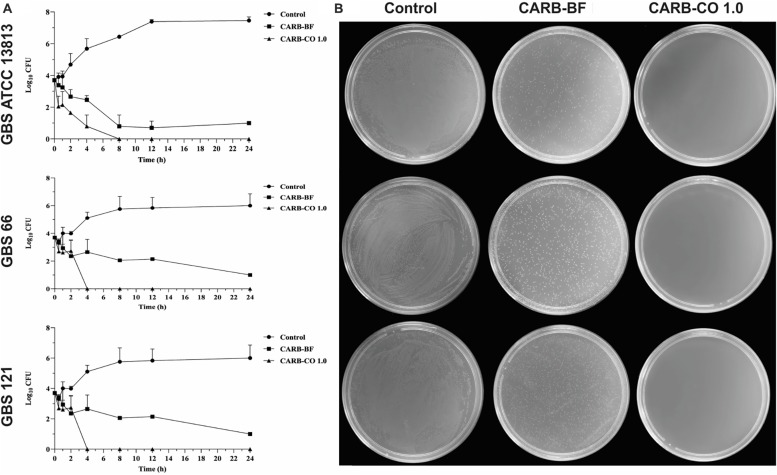
Antibacterial activity of carbomer-based hydrogels on growth of planktonic cells of *Streptococcus agalactiae*. **(A)** Time-kill curve of *S. agalactiae* ATCC 13813; erythromycin and clindamycin-resistant GBS 66 strain; and erythromycin-resistant GBS 121 strain in presence of hydrogels. Bacteria were incubated with CARB-BF and CARB-CO 1.0 for 24 h at 37°C and the CFU counts per 10 μL were determined at specified time points. GBSs without treatment were used as growth control. **(B)** TSA plate assay. Values are mean ± standard deviation of two experiments.

In this study, a carbomer polymer (Carbopol^®^ 940, 0.6% w/w) was used to prepare hydrogels containing 0.5% and 1.0% of copaiba oil (Table 2). In order to mimic the vaginal pH (around 3.5 to 4.5), citric acid was added to achieve the pH value of 4.5. Compared to the untreated cells (Figure 3), CARB-BF inhibited partially the growth of all tested strains in the TSA plate assay, indicating that the Carbopol^®^ 940 has inherent antibacterial activity. Similar to the CARB-BF inhibitory effect, a decrease in GBS growth was observed in presence of CARB-CO 0.5 (data not shown). CARB-CO 1.0 totally inhibited the visual growth of planktonic cells of the reference, GBS 66 and GBS 121 strains (Figure 3), preserving the bactericidal activity of copaiba oil. In addition to CARB-CO 1.0 pH 4.5, hydrogels with pHs 5.5 and 7.0 were also prepared to evaluate the pH effect on antibacterial activity of the formulations. CARB-CO 1.0 with these pHs also inhibited the growth of GBS strains in the TSA plate assay (Supplementary Figure S2). Thus, CARB-CO 1.0 pH 4.5 hydrogel was chosen for further assays.

The killing kinetic of GBS planktonic cells provoked by CARB-CO 1.0 hydrogel was monitored during 24 h-incubation (Figure 3). CARB-BF hydrogel inhibited the growth of all tested GBS, and around 2 log_10_ -decrease (*P* < 0.01) in CFU counts was observed for these strains compared to initial inoculum, corroborating the results of TSA plate. However, the addition of 1% copaiba oil into the carbomer-based hydrogel led to total GBS growth inhibition; no CFUs were detected after 4 h for GBS 66 strain, and 8 h for reference and GBS 121 strains. These data confirmed the bactericidal effect of CARB-CO 1.0 hydrogel against GBS strains, and this effect was observed in a shorter time compared to copaiba oil alone (Figures 1A–C).

**FIGURE 4:**
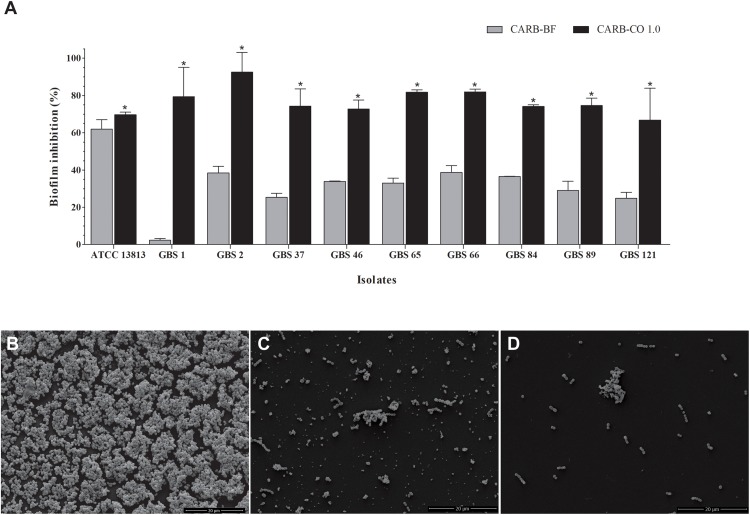
Inhibitory effect of carbomer-based hydrogels on GBS biofilm formation. **(A)** CARB-CO 1.0 and CARB-BF were spread on the surface of flat-bottom 24-well plates. Biofilm was formed during 24 h at 37°C and CFU counts were determined. Values are mean ± standard deviation of two experiments. The asterisks indicate a significant percentage reduction of biofilm on polystyrene surface previously loaded with CARB-CO 1.0 compared to untreated ones (^∗^*P* < 0.05). Scanning electronic microscopy images of biofilms formed on polystyrene surface for 24 h at 37^*o*^C: untreated surface **(B)**, previously loaded with CARB-BF **(C)** or CARB-CO 1.0 **(D)** for 24 h at 37°C. Bar: 20 μm.

**FIGURE 5:**
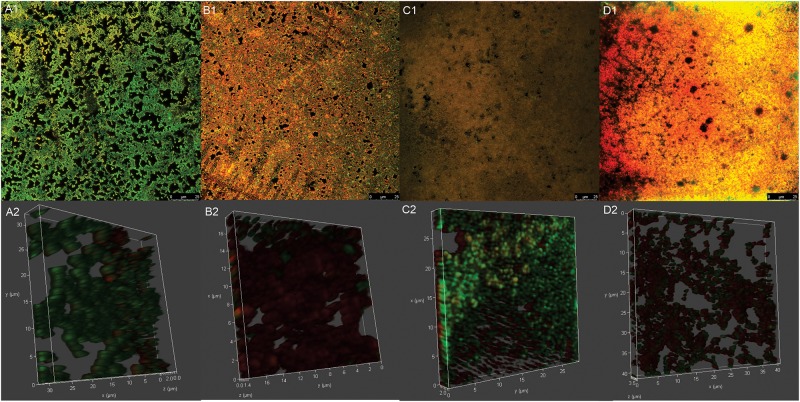
Confocal laser scanning microscopy images of mature biofilm of *Streptococcus agalactiae* ATCC 13813 untreated control **(A1,A2)** and treated with copaiba oil **(B1,B2)**, CARB-BF **(C1,C2)** and CARB-CO 1.0 **(D1,D2)**. Biofilms were formed on CELLview^TM^ cell culture dish with glass bottom during 24 h at 37°C before the treatments. Cells were stained with SYTO 9^®^ (green-fluorescent) and propidium iodide (red-fluorescent). Panoramic view of biofilm **(A1–D1)**. Three-dimensional biofilm reconstitution **(A2–D2)**.

The CARB-CO 1.0 hydrogel also preserved the antibiofilm activity in GBS strains. By using an *in vitro* prophylactic model, in which the hydrogels were loaded on the polystyrene surface before the bacterial inoculation, CARB-BF inhibited the biofilm formation with a percentage reduction in CFU counts of sessile cells ranging from 2.4 ± 0.9% to 62.0 ± 5.0%. The addition of copaiba oil at 1% to the hydrogel led to a significant reduction (*P* < 0.05) in the CFU counts in the biofilm when compared to the CARB-BF hydrogel. A percentage reduction ranging from 69.7 ± 1.3% to 92.5 ± 10.5% in CFU counts was observed for CARB-CO 1.0 hydrogel (Figure 4A). Consistent with these results, SEM images of GBS biofilms formed on polystyrene strips previously loaded with CARB-BF (Figure 4C), and CARB-CO 1.0 (Figure 4D) showed a significant decrease in the surface-adhered cells compared to the untreated control biofilm (Figure 4B). Moreover, a remarkable decrease in viability of sessile cells within the established biofilm of GBS strain was observed after treatment with both hydrogels (Figure 5). Indeed, as with copaiba oil (Figures 5B1,B2), CLSM images showed a high proportion of dead (red-fluorescent) cells in biofilm treated with CARB-BF (Figures 5C1,C2), while in CARB-CO 1.0 treated biofilm, the majority of the cells was dead (Figures 5D1,D2).

### Physical-Chemical Characteristics of CARB-CO 1.0 Hydrogel

The hydrogel containing 1.0% copaiba oil was slightly whitish in color, smooth in texture and presented slight turbidity visually when compared to CARB-BF, which was transparent. Despite this difference, CARB-CO 1.0 was homogeneous, and no phase separation was observed after centrifugation. Due to their high-water content, density-like water values of 1.0366 g/mL and 1.0658 g/mL were detected for CARB-BF and CARB-CO 1.0, respectively. The pH values of 5.48 and 5.75 were detected for CARB-BF and CARB-CO 1.0 hydrogels, respectively. As mentioned before, citric acid was added to achieve the pH value of 4.5, for maintenance of the physiological vaginal pH. These characteristics remained unchanged up to 6 months when kept at 4^*o*^C.

**FIGURE 6:**
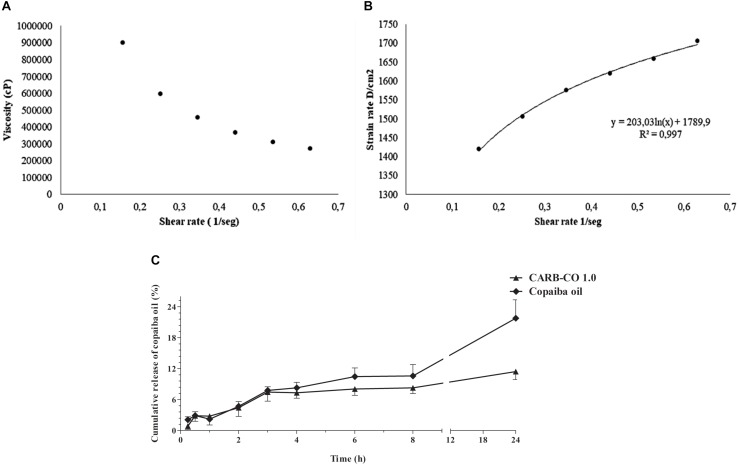
Rheological characteristics and *in vitro* release of *C. officinalis* oleoresin loaded into CARB-CO 1.0 hydrogel. Viscosity *versus* shear rate of CARB-CO 1.0 **(A)**. CARB-CO 1.0 rheogram **(B)**. Time-dependent release of copaiba oil from CARB-CO 1.0 **(C)**.

Viscosity analysis showed a non-Newtonian behavior of CARB-CO 1.0 hydrogel, which denotes a decrease in viscosity with a corresponding increase in shear rates (Figure 6A). According to the Ostwald-de Waele Power law model, the calculated *n*-value of hydrogel was lower than 1 (σ = k^∗^γ^*n*^), corroborating that CARB-CO 1.0 showed pseudoplastic profile (Figure 6B). This feature indicates an easy application of this hydrogel and maximum area coverage in topical use.

The *in vitro* profile of copaiba oil release from the CARB-CO 1.0 hydrogel was reported in Figure 6C. The copaiba oil showed a prolonged release under the test conditions, evidenced by the plateau formed after 4 h. At 4 and 8 h, the average percentage of released copaiba oil was 7.32% and 8.28%, respectively, corresponding to approximately 0.07 mg/mL and 0.08 mg/mL, which is consistent with the data generated by the time-kill kinetics at MBC values of the analyzed strains (Figure 1). At 24 h, the average percentage of released copaiba oil was 11.4%, corresponding to approximately 0.11 mg of copaiba oil, indicating a slow release, which was observed in the release kinetic constant with a value of 4.23/h. The release profile of copaiba oil *in natura* from the donor chamber was also evaluated to analyze the influence of the support (membrane) on oil diffusion. As observed in Figure 6C, the release of copaiba oil was faster than those observed for CARB-CO 1.0 hydrogel, mainly after 3 h, reaching an average percentage of diffusion of 8.30%, 10.60% and 21.80% in 4, 8 and 24 h, respectively.

The mechanism of copaiba oil release was analyzed by general release equation and an *n*-value of 0.28 was detected for CARB-CO 1.0, indicating that the release of the copaiba oil may involve a classical fickian diffusion-controlled drug release (Borghi-Pangoni et al., 2017).

### CARB-CO 1.0 Hydrogel Exhibits Biocompatibility With the Murine Cervicovaginal Mucosa

**FIGURE 7:**
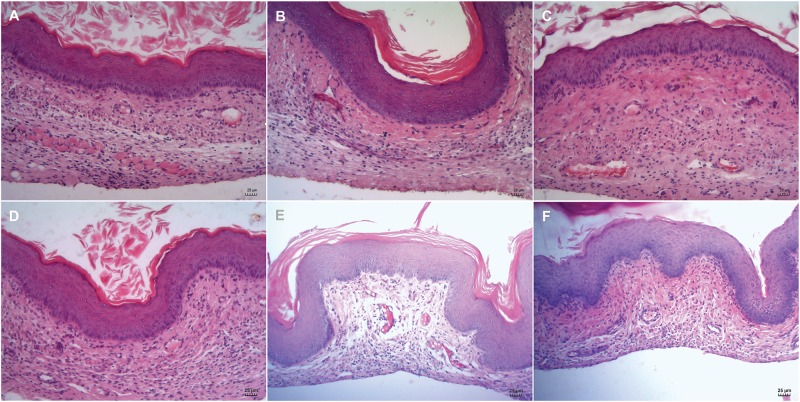
Biocompatibility of carbomer-based hydrogels. Histological analysis of cervicovaginal mucosa of BALB/c mice treated with PBS **(A,D)**, CARB-BF **(B,E)**, and CARB-CO 1.0 **(C,F)** after 24 h **(A–C)** and 14 days **(D–F)**. All images showed epithelial integrity and intact lamina propria. Bar: 25 μm.

The biocompatibility of the CARB-CO 1.0 hydrogel was evaluated directly on vaginal mucosa of BALB/c mice. After 24 h and 14 days of hydrogel application, cervicovaginal mucosa was surgically excised and prepared for histological analysis. The vaginal mucosa of untreated mice showed normal architecture and thickness, exhibiting well-defined epithelial layers with keratin deposition, and the lamina propria intact (Figures 7A,D). Similarly, no damage for vaginal epithelium and absence of inflammatory infiltrate after 24 h and 14 days of CARB-BF (Figures 7B,E) and CARB-CO 1.0 hydrogels (Figures 7C,F) application were observed.

## Discussion

The antibacterial activity of copaiba oil obtained from different *Copaifera* species against Gram-positive and Gram-negative bacteria has been previously reported in several studies (Santos et al., 2008a; Abrão et al., 2015; Tobouti et al., 2017; Diefenbach et al., 2018; Arruda et al., 2019). Thus, in attempt to find a natural product to overcome the potential adverse effects of IAP, our research group has previously described the inhibitory effect of oleoresin from *C. multijuga* Hayne on growth of planktonic and biofilm cells of GBS strains (Otaguiri et al., 2017). In the present study, we report the antibacterial activity of the oleoresin from *C. officinalis* Jacq. *in natura* (copaiba oil) and loaded into carbomer-hydrogel (CARB-CO 1.0) against GBS strains. As described for *C. multijuga*, oleoresin from *C. officinalis* provoked a dose- and time-dependent bactericidal effect against planktonic GBS strains presenting different susceptibility profiles to erythromycin and clindamycin. However, it seems that oleoresin from *C. officinalis* (MIC ranging from 0.03 to 0.06 mg/mL; MBC ranging from 0.06 to 0.12 mg/mL) has a more potent activity than that obtained from *C. multijuga* (MIC ranging from 0.12 to 0.50 mg/mL; MBC of ≥1.0 mg/mL). A more potent effect of *C. officinalis* oleoresin was also observed in biofilms of GBS strains (percentage reduction of metabolic activity ranged from 72.8% to 98.6% at 0.06 to 0.5 mg/mL), when compared to that of *C. multijuga* (percentage reduction of metabolic activity ranged from 23.7% to 60.6% at 1.0 mg/mL). Regardless of differences in inhibitory concentration values, the terpene components from *Copaifera* species appears to target the bacterial surfaces, affecting the cell wall, as well as the membrane integrity (Santos et al., 2008a; Otaguiri et al., 2017), as we also observed in this study.

The chemical compositions of oleoresin from different *Copaifera* species may vary according to several factors, including the genetic background of the plant, seasonality, and environmental conditions (Cascon and Gilbert, 2000; Santos et al., 2008a; Barbosa et al., 2012; Arruda et al., 2019); and as we pointed before the acid diterpenes appear to be responsible for most of the biological effects observed in oleoresin of different *Copaifera* species (Abrão et al., 2015; Vargas et al., 2015; Kian et al., 2018; Trindade et al., 2018; Arruda et al., 2019; Dalenogare et al., 2019). The sesquiterpene β-caryophyllene is largely found in both species (Santos et al., 2008b; Trindade et al., 2018; Arruda et al., 2019), and has been related to the anti-inflammatory activity of the *Copaifera* oil volatile fraction (Veiga Junior et al., 2007). Regarding acid diterpenes, the copalic acid (ranging from 1.1 to 11.0% of the total terpene content) was the main component in *C. multijuga* (Santos et al., 2008b; da Silva et al., 2017; Trindade et al., 2018); on the other hand, copalic (13.9% of the total terpene content), hardwickiic (30.7% of the total terpene content) and kaurenoic (13.8% of the total terpene content) acids have been reported in *C. officinalis* (Santos et al., 2008b; Dalenogare et al., 2019).

We also detected the presence of these three acid diterpenes in our study, and the kaurenoic acid appears to be one of the active components of oleoresin from *C. officinalis* related to antibacterial activity against GBS. In this sense, incorporation of the active component of copaiba oil in a pharmaceutical formulation will be a great option to overcome the fluctuating of the phytochemicals based on season and geographical location. However, the antibacterial activity observed in this study may not be attributed to only one, but several components of *C. officinalis* oleoresin acting synergistically, judging by the effect of kaurenoic acid on GBS biofilms. In fact, the antibacterial effect of copalic and kaurenoic acids extracted from *C. langsdorffii* oleoresin toward GBS ATCC 27591 strain was reported previously in the study of Abrão et al. (2015). Moreover, the purification of active component of copaiba oil will significantly increase the hydrogel cost, restricting its application, mainly in low-income or in developing countries.

A potential adverse effect associated with the use of antimicrobial compounds in the vaginal mucosa is the interference in its normal microbiota. *Lactobacillus* species are commonly found as commensal microorganisms of the human gastrointestinal tract and vagina (Hickey et al., 2012). These bacteria play an important role in protecting the vaginal ecosystem from invasive non-indigenous microorganisms, contributing to the maintenance of the vaginal pH. Many of the *Lactobacillus* species are susceptible to penicillin and ampicillin (Goldstein et al., 2015), which are the first-line antibacterials used in IAP for the control of GBS colonization (Verani et al., 2010). In this regard, our results showed that *C. officinallis* oleoresin was capable of killing GBS while had no effect on growth of *Lactobacillus* species, highlighting its potential for the development of novel pharmaceutical formulation for IAP. Supporting this, previous studies have shown that: (a) copaiba oil inhibited selectively the *in vitro* proliferation of human endometriotic stromal cells compared to endometrial normal cells (Henriques da Silva et al., 2015); (b) the topical vaginal cream containing copaiba oil did not cause toxicity in pregnant Wistar rats nor teratogenic effect on their offsprings (Lima et al., 2011).

The vaginal mucosa is a non-invasive route of drug delivery and presents some advantages such as ease of administration, high surface contact and bypass the first-pass metabolism. Due to these characteristics, this route has been explored for the delivery of locally acting drugs (Srikrishna and Cardozo, 2013). The topical use of copaiba oil in ointment is described in the Brazilian Pharmacopeia and is indicated as anti-inflammatory, antiseptic, and healing (Brazil Agência Nacional de Vigilância Sanitária, 2010). In fact, copaiba oil has been traditionally used orally *in natura* or topically as an ointment for the treatment of various diseases, including infections of the genitourinary tract (Leandro et al., 2012; Trindade et al., 2018). Moreover, copaiba oil is extracted directly from *Copaifera* tree by tapping its trunk with a manual metal auger, representing a renewable source of biologically active components. In view of these, and due to the results obtained with *C. officinalis* oleoresin, we developed a biocompatible, eco-friendly and low-cost copaiba oil-containing carbomer hydrogel, which preserved the antibacterial activity against GBS cells. This hydrogel exhibited potent bactericidal effect *in vitro*, killing both planktonic cells (in a shorter time compared to the effect of copaiba oil *in natura*) and sessile cells within the established biofilms, as well as inhibiting the biofilm formation by GBS strains susceptible and resistant to erythromycin and/or clindamycin. Furthermore, this hydrogel showed good biocompatibility with the vaginal mucosa of BALB/c mice, indicating safety for *in vivo* application. These characteristics are clinically important since the microbial biofilm is the mode of growth predominantly found during colonization and infection of host tissues (Donlan, 2002; Hardy et al., 2017). Microbial biofilms are communities of surface-attached microorganisms embedded in a self-produced extracellular polymeric matrix. Compared to their planktonic counterpart cells, biofilms are remarkably less susceptible to antimicrobial agents, contributing to their persistence in host environment. Crucially, most antimicrobial agents available for the treatment of infections does not present antibiofilm activity (Venkatesan et al., 2015).

Carbomers, which are high molecular weight cross-linked polymers of acrylic acid, have been commonly used for the preparation of semi-solid or liquid formulations for topical administration (de Araújo Pereira and Bruschi, 2012; Berretta et al., 2013; Hayati et al., 2018). The carbomer used in this study (Carbopol^®^ 940) is composed of polyacrylic acid subunits highly cross-linked with pentaerythritol allyl ethers, and due to the presence of hydroxyl and carboxyl groups, it exhibits an anionic characteristic. The flow rheological properties of the vaginal formulations are important factors that determine the ease of both the administration and the retention of the product in the mucosal membrane. In this study, the CARB-CO 1.0 hydrogel was homogeneous, soft, showed viscoelasticity, and hence good spreadability. In this sense, after exposure to shear stress, the dispersion can flow and the polymer chains of hydrogel can align along the shear direction, releasing water or water and copaiba oil. In addition, the release pattern of copaiba oil loaded into the CARB-CO 1.0 showed a slow and sustained release of the *C. officinalis* oleoresin. These characteristics can extend its residence time into the vaginal mucosa and control the rate of copaiba oil release (de Araújo Pereira and Bruschi, 2012). In fact, the pseudoplastic behavior of the 1% Carbopol^®^ 940-based hydrogels for vaginal application was described previously (Berretta et al., 2013). Importantly, this characteristic was not modified after addition of propolis (1%) and essential oils of *Betula lenta*, *Melaleuca alternifolia*, *Mentha spicata* and *Rosmarinus officinalis*, and propylene glycol. The Carbopol^®^ 940-containing propolis hydrogel was capable of reducing *Candida albicans* load on vaginal mucosa of BALB/c mice, exhibiting a good mucoadhesive property in absence of irritation or inflammation.

Besides the study of Berretta et al. (2013), others also proposed the design of carbomer-based hydrogels with antimicrobial activity for topical administration to the vaginal mucosa, most of which reported the inhibitory effect only on growth of planktonic bacterial cells. Xu et al. (2013) developed a polycarbophil/Carbopol^®^ 934R hydrogel containing benzoyl peroxide, an organic peroxide, which exhibited an *in vitro* bactericidal activity against *Gardnerella vaginalis*, a causative agent of bacterial vaginosis, without affecting the growth of species of *Lactobacillus*. Moreover, Esposito et al. (2013) designed a carbomer (Carbopol^®^ 974P NF) hydrogel loaded with clotrimazole encapsulated in monoolein aqueous dispersion nanosystem. Compared to the inhibitory effect of clotrimazole alone, this hydrogel led to a significant reduction of *C. albicans* growth *in vitro*. Singh et al. (2014) developed a bigel containing carbomer (Carbopol^®^ 934) and SMS-sesame oil forming an aqueous and apolar phase, respectively, loaded with metronidazole for the treatment of bacterial vaginosis. The bigel was viscoelastic in nature, showed biocompatibility with HaCaT keratinocyte cell line and antibacterial activity against planktonic cells of *Escherichia coli*.

In conclusion, our study reports the bactericidal and antibiofilm activities of oleoresin from *C. officinalis*, alone or incorporated into carbomer-hydrogel (CARB-CO 1.0), toward *S. agalactiae*. The hydrogel showed good biocompatibility with the murine vaginal mucosa and exhibited a viscoelastic behavior with slow and sustained release of the *C. officinalis* oleoresin. These results indicate that carbomer-based hydrogels may be useful as a topical and mucoadhesive system for delivery of copaiba oil directly into the vaginal mucosa for controlling *S. agalactiae* colonization and infection. Further *in vivo* studies will determine the effectiveness and safety of the CARB-CO 1.0 hydrogel.

## Data Availability Statement

All datasets generated for this study are included in the article/Supplementary Material.

## Ethics Statement

This study protocol was approved by the Ethics Committee for the Use of Animals of Universidade Estadual de Londrina (Document 127/2017-CEUA/UEL) and by the Ethics Committee of UEL (Document 193/12-CEP/UEL).

## Author Contributions

All authors listed have made a substantial, methodological, and intellectual contribution to the study. AM and SY-O performed the conception, experimental design, analysis and interpretation of data, and writing of the manuscript. All authors have read and approved the final manuscript.

## Conflict of Interest

The authors declare that the research was conducted in the absence of any commercial or financial relationships that could be construed as a potential conflict of interest.

## Supplementary Material

FIGURE S1 Representative mass spectra profile of acid diterpenes (copalic, hardwickiic and kaurenoic acids) from *Copaifera officinalis* oleoresin.

FIGURE S2 Antibacterial activity of carbomer-based hydrogels with different pHs on growth of *S. agalactiae* ATCC 13813. The bacteria were incubated with CARB-BF **(B,C)** and CARB-CO 1.0 **(D,E)**, in pHs 5.5 **(B,D)** and 7.0 **(C,D)** during 24 h at 37^*o*^C. GBSs without treatment **(A)** were used as growth control.
