# Supplementary figures and images for: Hydrogel Containing Oleoresin From Copaifera officinalis Presents Antibacterial Activity Against Streptococcus agalactiae
Source: Front Microbiol. 2019 Dec 4;10:2806. doi: 10.3389/fmicb.2019.02806 (PMC6904337; doi:10.3389/fmicb.2019.02806)

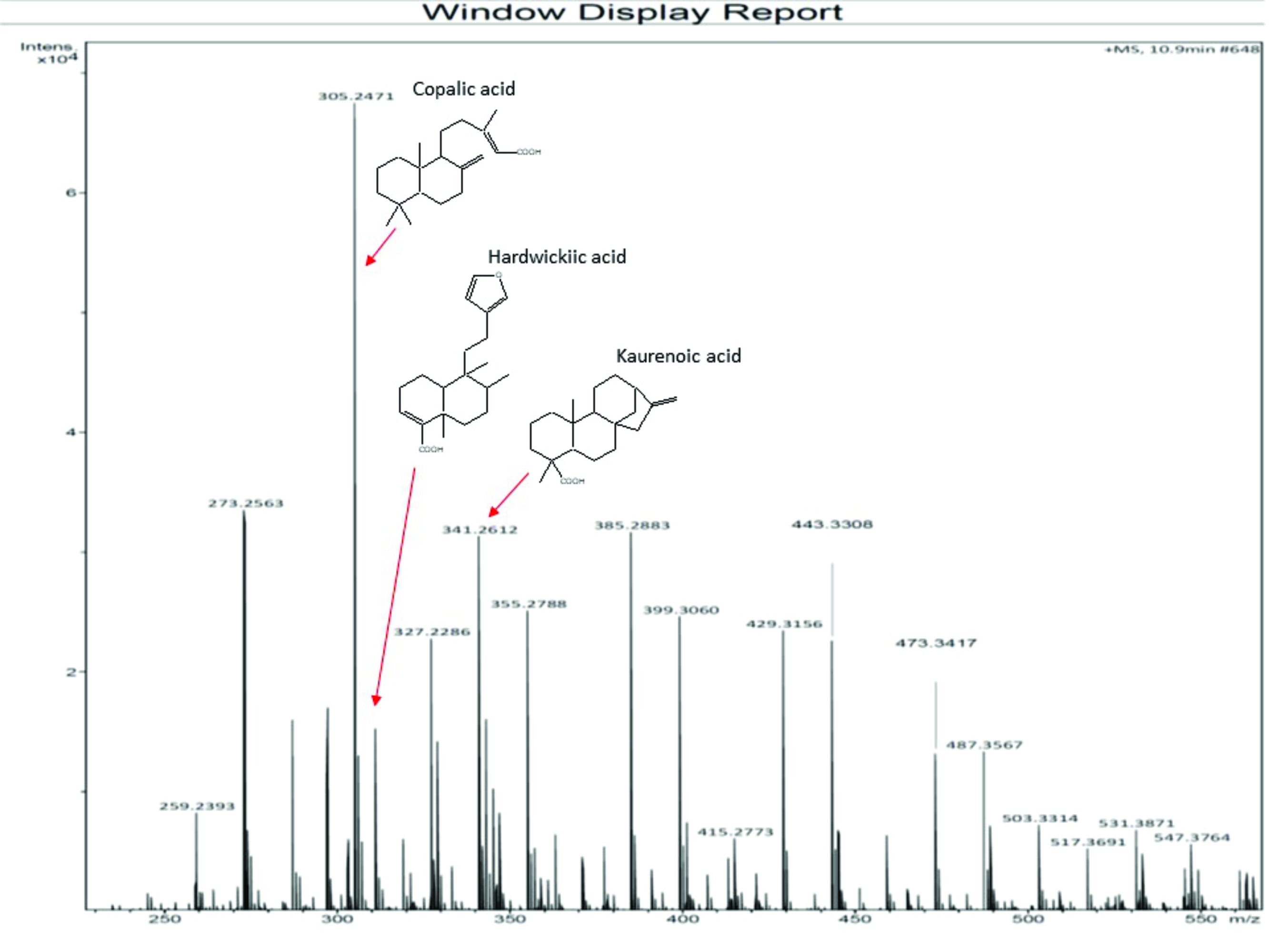

Supplement: FIGURE S1 — Representative mass spectra profile of acid diterpenes (copalic, hardwickiic and kaurenoic acids) from Copaifera officinalis oleoresin. [file Image_1.TIF]

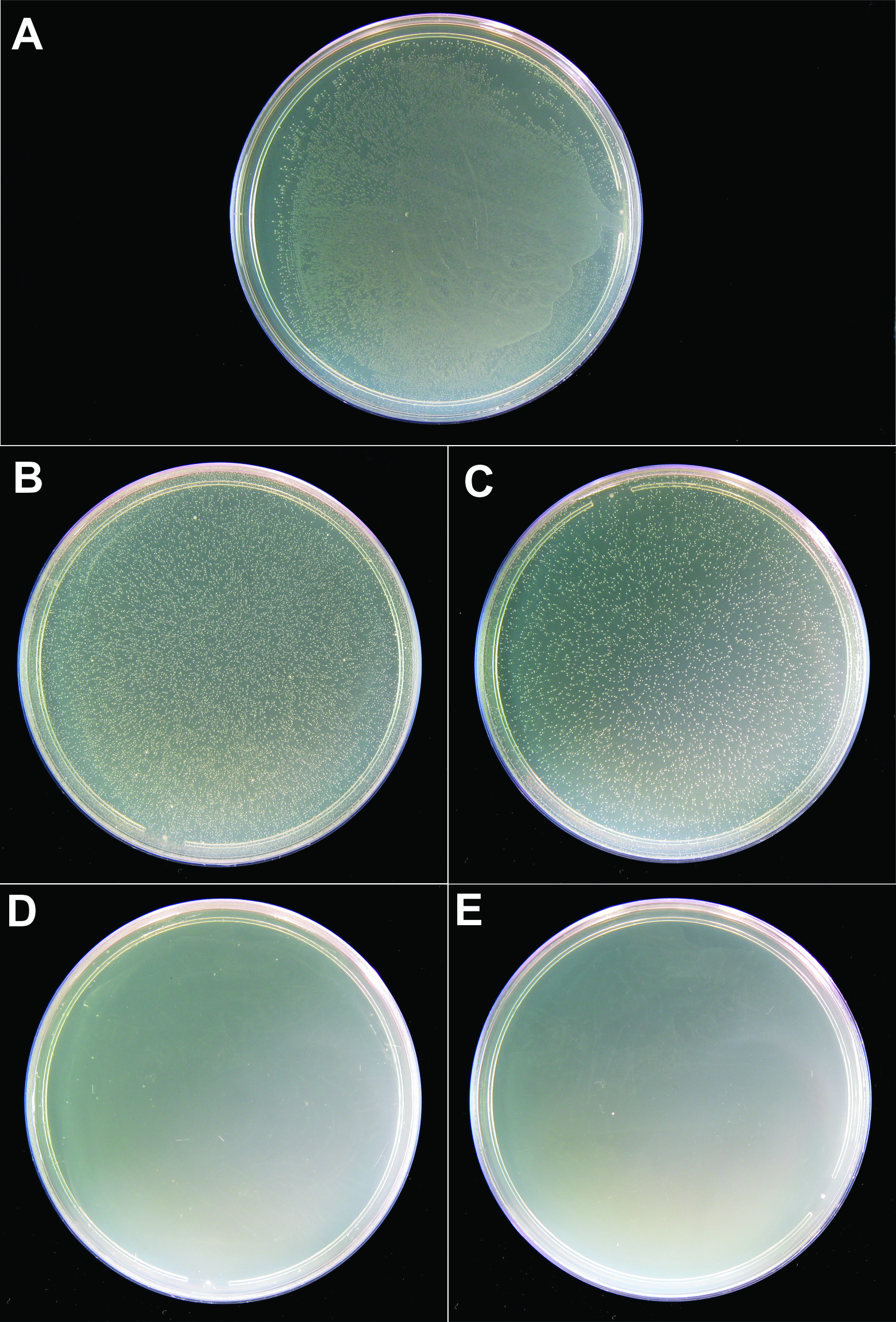

Supplement: FIGURE S2 — Antibacterial activity of carbomer-based hydrogels with different pHs on growth of S. agalactiae ATCC 13813. The bacteria were incubated with CARB-BF (B,C) and CARB-CO 1.0 (D,E), in pHs 5.5 (B,D) and 7.0 (C,D) during 24 h at 37oC. GBSs without treatment (A) were used as growth control. [file Image_2.TIF]
